# A Prospective Analysis of Risk Factors and Outcomes of Intracranial Atherosclerosis

**DOI:** 10.7759/cureus.86221

**Published:** 2025-06-17

**Authors:** Vaiyapuri Balambighai, Neeraja Ponnala, Stephen Sureshkumar

**Affiliations:** 1 Neurology, Sree Balaji Medical college and Hospital, Chennai, IND; 2 Neurology, Sree Balaji Medical College and Hospital, Chennai, IND

**Keywords:** intracranial atherosclerosis, ischemic stroke, magnetic resonance angiography, stenosis, stroke recurrence

## Abstract

Introduction: Intracranial atherosclerosis (ICAS) is a progressive disease that leads to narrowing of the brain’s arteries and reduced blood flow, making it a leading cause of both initial and recurrent strokes worldwide. Various contributing factors influence its development. Despite advanced neuroimaging and therapeutic interventions, the management of ICAS remains a concern. Our study examines the risk factors and outcomes of ischemic stroke due to ICAS in a tertiary care facility.

Methods: This prospective study examines 100 patients with ICAS-related acute ischemic stroke. Diagnosis was established by magnetic resonance angiography (MRA). Clinical assessments, imaging studies, laboratory tests, and follow-up evaluations were performed over a 12-month period. Recurrence of stroke and function status were evaluated using multivariate Cox regression analysis and Kaplan-Meier survival curves.

Results: Dominant risk factors were hypertension, seen in 82 (82%) patients, followed by diabetes mellitus, which was seen in 52 (52%) patients. Smoking as a risk factor was reported in 34 (34%) cases, followed by hyperlipidemia, which was seen in 29 (29%) cases. The middle cerebral artery was involved in 53 (53%) patients. Stroke recurrence occurred in 17 (17%) patients within the first three months. The independent presence of three or more risk factors raised recurrence risk (p=0.035). Dual antiplatelet and statin therapies significantly reduced recurrence (p<0.05). Smoking and higher National Institutes of Health Stroke Scale (NIHSS) scores were linked to worse functional outcomes.

Conclusion: This study confirms a greater prevalence of intracranial atherosclerotic stroke among men. Intensive medical treatment, such as incorporating dual antiplatelet therapy, statins, and strict management of diabetes and hypertension, showed a significant reduction in recurrence rates.

## Introduction

Ischemic stroke has been one of the major contributors to global mortality in recent times [1,2]. In intracranial atherosclerosis (ICAS) cases, atherosclerotic plaque accumulation leads to progressive stenosis of large intracranial arteries [3]. It has been frequently linked to higher rates of stroke recurrence and, therefore, has been proposed as an important target for early diagnosis and intervention [4]. Although there has been significant development in neuroimaging technologies and treatments, ICAS remains a major threat to world health [5]. Asian, Hispanic, and African subjects displayed a comparatively higher incidence of ICAS [3,6].

ICAS is characterized by endothelial dysfunction, chronic inflammation, and lipid deposition, leading to progressive arterial stenosis and compromised cerebral perfusion [7]. The affected arteries are usually intracranial branches of the internal carotid artery, middle cerebral artery, anterior cerebral artery, posterior cerebral artery, basilar artery, and vertebral artery [8]. Stenosis can range from mild vessel wall thickening to severe occlusive plaques, with thrombus formation elevating the risk of stroke. The mechanisms underlying stroke in ICAS include in situ thrombosis, artery-to-artery embolism, local perforator occlusion, and hemodynamic failure due to compromised collateral circulation [9].

Epidemiological studies have shown significant geographic and racial variations in the prevalence of ICAS. The burden is notably higher in South Asian populations, with rates ranging from 33% to 50% among individuals presenting with acute ischemic stroke [1,10]. Similar trends have also been observed in African American as well as Hispanic subjects, in whom ICAS is a common cause of stroke in terms of extracranial carotid artery disease [11]. The condition is also more prevalent in men as compared with women, particularly in those in mid-life. The progression of ICAS is greatly affected by classical atherogenic risk factors, which are hypertension, diabetes mellitus, dyslipidemia, metabolic syndrome, as well as cigarette smoking [12]. Age, genetic background, as well as race are non-modifiable risk factors that also play a role in making a person susceptible to the condition [13].

Neuroimaging has improved the detection and recognition of intracranial atherosclerotic stenosis (ICAS). Digital subtraction angiography (DSA) remains considered the gold standard of evaluation of the degree of stenosis [14]. However, the use of non-invasive imaging methods like high-resolution magnetic resonance angiography (MRA), computed tomography angiography (CTA), and transcranial Doppler ultrasound (TCD) is becoming more common in clinical practice [7,15]. New imaging technologies like vessel wall MRI provide informative information on plaque vulnerability and recurrent stroke risk occurrence [16]. Furthermore, biomarkers like high-sensitivity C-reactive protein (hs-CRP), plasminogen activator inhibitor-1, and lipoprotein-associated phospholipase A2 (Lp-PLA2) have also been studied for their role in the prediction of disease progression and recurrent stroke occurrence [17,18].

Management of ICAS is directed towards optimal medical therapy, lifestyle change, and, in certain situations, endovascular treatment. The pillars of secondary stroke prevention are dual antiplatelet therapy, intensive lipid-lowering therapy, and strict blood pressure and blood glucose control [19,20]. Clinical trials have also contrasted the effectiveness of anti-inflammatory drugs and new lipid-lowering drugs in the prevention of vascular events [5]. Endovascular procedures like intracranial angioplasty and stenting have been recommended for patients with high-grade stenosis who remain at risk for ischemic events despite optimal medical therapy [21]. Randomized controlled trials have, however, questioned the safety and long-term efficacy of such procedures.

Despite extensive research, several lacunae remain in identifying determinants of stroke recurrence in ICAS, the predictive role of new biomarkers, and the role of new imaging tools in therapeutic decision-making. Additionally, the synchrony between medical and interventional approaches in high-risk patients remains poorly elucidated. Therefore, the current study explores the clinical profile, risk factors, and outcomes of ICAS patients with ischemic stroke in a tertiary care center. Based on imaging patterns and recurrence rates, the study will identify predictors of adverse outcomes and assess the role of varying management strategies. The results of the study may enhance risk stratification and maximize treatment strategy in ICAS in clinical practice.

## Materials and methods

Study design and setting

The patients with acute ischemic stroke attributable to ICAS were included in this prospective study during the course of a year, from August 2021 to July 2022, which was carried out at a tertiary care medical college hospital in Chennai, India. A set of inclusion and exclusion criteria led to the enrollment of 100 patients in total. The institutional ethics committee gave its approval to the study, and each participant gave their informed consent.

Study population

Patients aged 40 to 70 years who were presented with a transient ischemic attack (TIA) or ischemic stroke within seven days of symptom onset were included. Diagnosis of intracranial large artery occlusion was confirmed using magnetic resonance angiography (MRA) following the TOAST classification (≥50% stenosis of a major intracranial artery). Exclusion criteria included patients with extracranial internal carotid artery stenosis, cardioembolic stroke, severe systemic illnesses, or those who could not undergo MRA or computed tomography angiography (CTA) due to contraindications.

Clinical and imaging assessment

Comprehensive clinical and imaging assessments were performed. Demographic details, vascular risk factors, clinical presentation, neurological examination findings, laboratory investigations, and treatment history were recorded in a structured case report form. Neuroimaging was performed using 3D time-of-flight (TOF) MRA on a 3-Tesla MRI scanner (Waukesha, WI: GE Healthcare). For patients for whom MRA was contraindicated, computed tomography angiography (CTA) was used as an alternative. A stenosis or occlusion of at least 50% of the major intracranial arteries, such as the basilar artery, the intracranial segments of the internal carotid and vertebral arteries, and the proximal segments of the middle cerebral, anterior, and posterior cerebral arteries, was considered significant intracranial atherosclerotic disease. Intracranial atherosclerotic disease is defined as a 50-99% diameter stenosis of a major intracranial artery due to atherosclerotic lesions. Stenosis was detected by MRA, CTA, or catheter angiography. Stenosis was graded as mild (<50%), moderate (50-69%), severe (70-99%), and complete occlusion (100%), based on Society of Cardiovascular Computed Tomography (SCCT) recommendations [12]. The location, severity, and distribution of stenotic lesions were systematically documented. Additional laboratory investigations included fasting blood glucose, lipid profile, homocysteine levels, and inflammatory markers such as C-reactive protein (CRP). Electrocardiography (ECG) and echocardiography were conducted to exclude cardioembolic sources.

Follow-up and outcome assessment

All patients were followed for one year, with routine outpatient visits scheduled at one, three, six, and 12 months. Follow-up assessments focused on secondary stroke prevention strategies, including management of vascular risk factors, lifestyle modifications, and adherence to antithrombotic and lipid-lowering therapies. Functional status was evaluated using the Modified Rankin Scale (mRS), and recurrence of stroke or other vascular events was recorded. Drug compliance was monitored through patient interviews and prescription refill records. The primary outcome was the time to first recurrent ischemic stroke within one year. Secondary outcomes included any major vascular events, such as TIA, hemorrhagic stroke, acute coronary syndrome, or other stroke-related complications. Patients with mRS scores of ≥3 or mortality were categorized as having unfavorable outcomes.

Statistical analysis

Risk factors and baseline characteristics were gathered using descriptive statistics. Categorical variables were presented as percentages and frequencies, while continuous variables were presented as means with standard deviations. Depending on the data distribution, the Student's t-test or Mann-Whitney U test was used for continuous variables, and the Chi-square test or Fisher's exact test for categorical variables for comparing groups. Cox proportional hazards regression models were used in multivariate analysis to find stroke recurrence predictors. The multivariate model contained variables whose p-values in univariate analysis were less than 0.10. The cumulative risk of recurrent stroke was estimated using Kaplan-Meier survival analysis, and survival curves were compared using log-rank testing.

Sensitivity analyses were performed to account for potential biases due to loss of follow-up, and adjustments were made for confounders such as sex, age, and severity of initial stroke. P-values less than 0.05 were regarded as statistically significant. For all statistical studies, SPSS software version 26.0 (Armonk, NY: IBM Corp.) was used. This study provides an evaluation of intracranial atherosclerosis in acute stroke patients, emphasizing risk factors, imaging characteristics, treatment outcomes, and statistical modeling of recurrence and prognosis over a one-year period.

## Results

Patient characteristics and risk factors

A total of 100 patients diagnosed with acute ischemic stroke due to intracranial atherosclerosis were included in this study. Among them, 68 were male and 32 were female. The mean age of stroke onset was 54.88±12.2 years in males and 55.97±11.2 years in females. Hypertension was the most prevalent risk factor, present in 82 (82%) patients, with slightly higher rates in males compared to females (57 {83.8%} in males versus 25 {78.1%} in females). Diabetes was identified in 36 (52.9%) males and 16 (50.0%) females. Hyperlipidemia was found in 18 (26.5%) males and 10 (31.3%) females. Homocysteinemia was present in 15 (22.1%) males and eight (25.0%) females. Among lifestyle-related risk factors, smoking was reported in 31 (45.6%) males and two (6.3%) females, while alcohol consumption was noted in 31 (45.6%) males and three (9.4%) females (Table 1).

**Table 1:** The baseline characteristics and risk factors of the patients. DM: diabetes mellitus

Variables	Male (n=68)	Female (n=32)
Age (mean±SD)	54.88±12.2	55.97±11.2
Alcohol, n (%)	31 (45.6)	3 (9.4)
DM, n (%)	36 (52.9)	16 (50.0)
Homocysteine, n (%)	15 (22.1)	8 (25.0)
Hypertension, n (%)	57 (83.8)	25 (78.1)
Hyperlipidemia, n (%)	18 (26.5)	10 (31.3)
Smoking, n (%)	31 (45.6)	2 (6.3)
Others, n (%)	7 (10.3)	3 (9.4)

Distribution of arterial occlusion and associated risk factors

Hypertension was common across all arterial lesions, with the highest prevalence in 27 (93.1%) patients with posterior cerebral artery (PCA) involvement. Diabetes was strongly associated with basilar artery (BA) occlusion, seen in 10 (83.3%) patients, and vertebral artery (VA) stenosis, which was seen in 14 (70.0%) patients. Hyperlipidemia was most frequently found in six (50%) patients with BA stenosis and in eight (40%) patients with VA involvement. Homocysteinemia was observed in 17 (28.7%) cases with middle cerebral artery (MCA) stenosis. Smoking and alcohol use were more frequent in patients with MCA and internal carotid artery (ICA) occlusions compared to other arterial territories, as presented in Table 2.

**Table 2:** The sites of arterial occlusion and the relation to risk factors. All the figures are in percentage points. HTN: hypertension; DM: diabetes mellitus; MCA: middle cerebral artery; ICA: internal carotid artery; ACA: anterior cerebral artery; VA: vertebral artery; BA: basilar artery; PCA: posterior cerebral artery

Variables	MCA	ACA	ICA	VA	BA	PCA
HTN, n (%)	47 (79.6)	11 (84.6)	21 (77.8)	16 (80.0)	9 (75.0)	27 (93.1)
DM, n (%)	27 (44.2)	2 (15.4)	12 (44.4)	14 (70.0)	10 (83.3)	16 (55.2)
Hyperlipidemia, n (%)	17 (28.6)	1 (7.7)	6 (22.2)	8 (40.0)	6 (50.0)	11 (37.9)
Homocysteinemia, n (%)	17 (28.7)	2 (15.4)	8 (29.6)	3 (15.0)	2 (16.7)	4 (13.8)
Alcohol, n (%)	22 (38.1)	3 (23.1)	13 (48.1)	9 (45.0)	5 (41.7)	7 (24.1)
Smoking, n (%)	23 (39.5)	6 (46.2)	14 (51.9)	6 (30.0)	5 (41.7)	11 (37.9)
Others, n (%)	1 (3.2)	2 (15.4)	-	5 (25.0)	1 (8.3)	5 (17.2)

Symptoms based on the circulation territory

**Figure 1:**
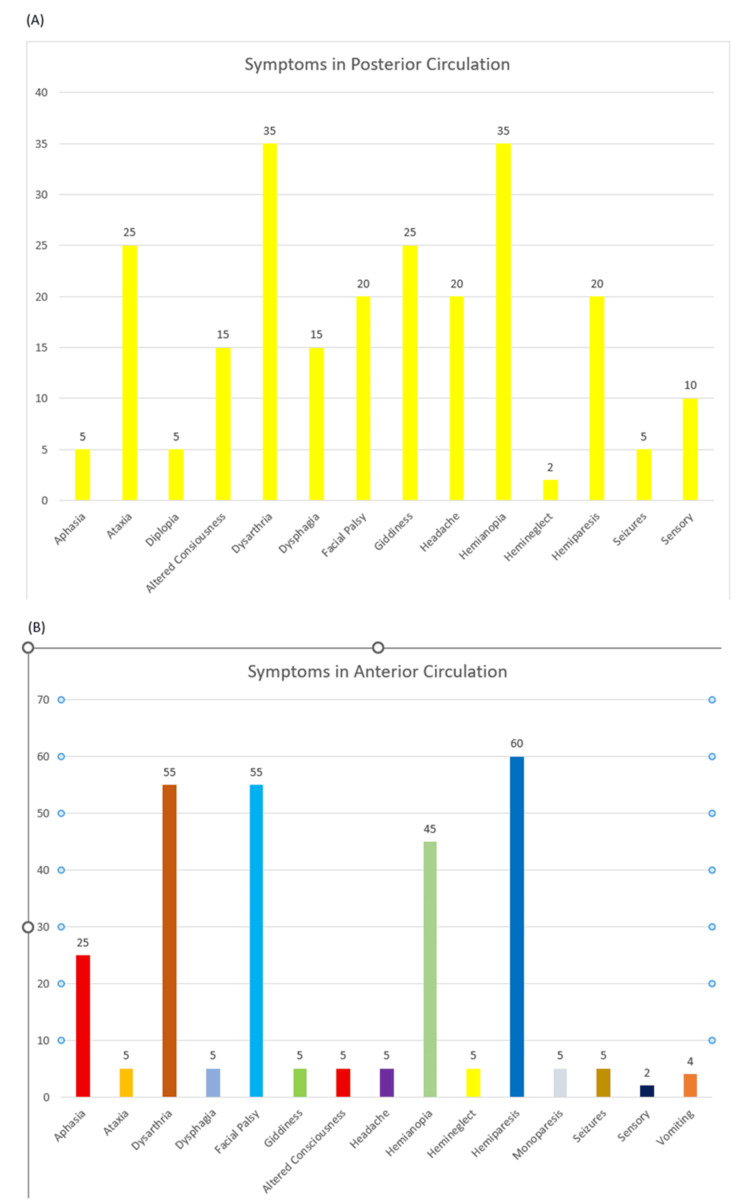
Symptoms in posterior and anterior circulation. Symptoms associated with posterior and anterior circulation strokes: (A) distribution of symptoms in patients with posterior circulation strokes, showing the frequency of common clinical manifestations such as dysarthria, hemianopia, hemiparesis, and altered consciousness; (B) symptoms observed in anterior circulation strokes, highlighting lower limb weakness, giddiness, aphasia and hemianopia as predominant features.

**Figure 2:**
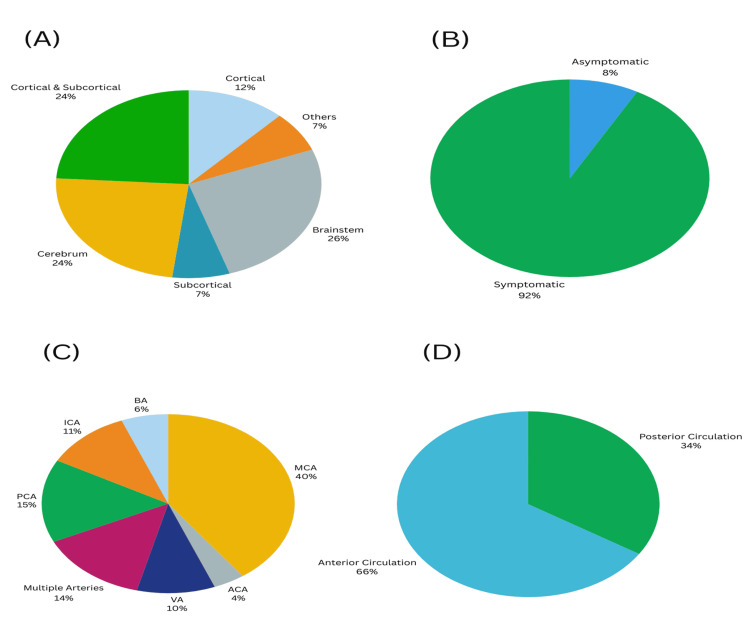
Distribution and characteristics of intracranial atherosclerotic lesions and infarcts. (A) Topographical distribution of infarcts, (B) frequency of symptomatic and asymptomatic lesions, (C) the percentage distribution of intracranial atherosclerotic lesions in various intracranial arteries, and (D) distribution of lesions according to anterior circulation and posterior circulation. MCA: middle cerebral artery; ICA: internal carotid artery; ACA: anterior cerebral artery; VA: vertebral artery; BA: basilar artery; PCA: posterior cerebral artery

In posterior circulation strokes, the leading symptoms were dysarthria, hemianopia, ataxia, and giddiness (Figure 1, panel A). The most common symptoms in anterior circulation strokes included hemiparesis, dysarthria, and headache (Figure 1, panel B). Infarcts were distributed across various brain regions, with the cerebral cortex affected in 26 patients, both cortical and subcortical regions in 24 patients, and in 24 patients, it was confined to the subcortical areas. Cerebellar infarcts accounted for 12%, while brainstem infarcts and other locations each constituted 7% of cases (Figure 2, panel A). Among the 100 patients, eight (8%) had asymptomatic intracranial lesions (Figure 2, panel B).

Frequency and location of atherosclerotic lesions

Among the affected arteries, the middle cerebral artery (MCA) was the most frequently involved, observed in 40 patients (40%), followed by the posterior cerebral artery (PCA), seen in 15 patients (15%). ICA is involved in 11 (11%) patients, followed by VA involvement in 10 (10%) patients. Anterior cerebral artery is involved in six (6%), and BA was the site of arterial occlusion in four (4%) patients (Table 3 and Figure 2, panel C). In 14 (14%) cases, multiple vessels were involved, with combinations such as ICA+MCA+ACA, BA+PCA, and VA+BA. The number of patients with anterior circulation stroke was 66 (66%), whereas 34 (34%) patients had posterior circulation involvement (Figure 2, panel D).

**Table 3:** Frequency of atherosclerotic lesions in various arteries. MCA: middle cerebral artery; ICA: internal carotid artery; ACA: anterior cerebral artery; VA: vertebral artery; BA: basilar artery; PCA: posterior cerebral artery

Artery	MCA	ICA	ACA	VA	BA	PCA	MCA+ICA	ICA+MCA+ACA	BA+PCA	VA+BA
Frequency (%)	40	11	4	10	6	15	4	4	4	2

Recurrence of stroke and risk factors

During the follow-up period, 17 patients experienced recurrent ischemic events, including stroke or transient ischemic attacks (TIA). MCA territory recurrence was most frequent (seven patients), followed by ICA (four patients) and vertebrobasilar circulation (six patients). Among these, 16 patients experienced recurrence in the initially affected stenotic territory. Non-lacunar infarcts were predominant in recurrent cases, with 12 of 17 recurrences occurring within the first three months post-stroke.

Predictors of stroke recurrence

Multivariate analysis showed that age, gender, hypertension, diabetes, hyperlipidemia, homocysteinemia, smoking, and alcohol use did not individually predict recurrence (Table 4). However, the presence of three or more risk factors significantly increased the likelihood of stroke recurrence (p=0.0351). Patients with multiple atherosclerotic lesions (>3 affected arteries) also had a higher recurrence risk. Patients presenting with vulnerable intracranial stenosis detected by high-resolution vessel wall MRI imaging features like plaque surface irregularity, intraplaque hemorrhage, high lipid volume, and contrast enhancement are also at risk of higher stroke recurrence.

**Table 4:** Predictors of recurrence (multivariate analysis). HTN: hypertension; DM: diabetes mellitus; NIHSS: National Institutes of Health Stroke Scale

Factors	No recurrence (n=83)	Recurrence (n=17)	Hazard ratio	p-Value
Age (mean±SD)	54.93±11.3	56.71±14.2	1.078	0.5075
Gender, n (%)	55 (66.3)	13 (76.5)	0.759	0.9048
HTN, n (%)	68 (81.9)	14 (82.4)	0.550	0.8359
DM, n (%)	41 (49.4)	11 (64.7)	7.347	0.6757
Hyperlipidemia, n (%)	20 (24.1)	8 (47.1)	0.044	0.0533
Alcohol, n (%)	25 (30.1)	9 (52.9)	0.582	0.7582
Smoking, n (%)	26 (31.3)	7 (41.2)	0.217	0.5391
Homocystinemia, n (%)	19 (22.9)	4 (23.5)	2.994	07390
Multiple lesions (>3), n (%)	32 (38.6)	7 (41.2)	8.753	0.4689
NIHSS (mean±SD)	10.64±4.3	12.8±4.6	1.076	0.5614
Risk factors (≥3), n (%)	36 (43.4)	14 (82.4)	218.294	0.0351

Impact of treatment and risk factor control on recurrence

Treatment adherence played a role in reducing recurrence. Table 5 shows that patients who complied with dual antiplatelet therapy had a lower recurrence rate (p<0.05) compared to those on monotherapy (p=0.004). Statin use was associated with better stroke prevention outcomes (p=0.016). Poorly controlled hypertension (p<0.05) and diabetes (p<0.05) were significant contributors to recurrence, while dyslipidemia control was not statistically associated with recurrence risk.

**Table 5:** Recurrence of stroke and the effect of treatments and uncontrolled risk factors. This table shows the factors associated with the recurrence of stroke in this study. As presented in the table, 17 patients had recurrent episodes of stroke, while there was no recurrence in 83 patients. Most of the patients in both groups were compliant with antiplatelets (90% and 76%, respectively). Treatment with dual antiplatelets and high-intensity statins has shown a good effect in preventing another stroke (p<0.05). Those who were on oral monotherapy with platelet antagonists were noted to have a higher chance of recurrence compared to those on dual combination therapy (p=0.004). The presence of uncontrolled hypertension (64% in the recurrence group) and diabetes mellitus (35% in the recurrence group) was significantly associated with higher stroke recurrence in our study (p<0.05). HTN: hypertension; DM: diabetes mellitus

Factors	No recurrence (n=83)	Recurrence (n=17)	p-Value
Compliance with antiplatelets	75/83 (90%)	13/17 (76%)	0.1
Dual antiplatelet Therapy	70/83 (84%)	6/17 (35%)	<0.05
Single antiplatelet therapy	5/83 (6%)	7/17 (41%)	0.004
Statin therapy	70/83 (84%)	10/17 (59%)	0.016
Patients with poor glycemic control	6/41 (15%)	4/11 (35%)	<0.05
Patients with poor BP control with drugs	8/68 (12%)	9/14 (64%)	<0.05

Outcome at thee, six, and 12 months

At the three-month follow-up, 56 patients had a favorable outcome, while 44 had an unfavorable outcome. Smoking was significantly associated with worse outcomes (p=0.047). Hemiparesis was observed more frequently in those with unfavorable outcomes (p=0.0086), and higher National Institutes of Health Stroke Scale (NIHSS) scores at admission were linked to poorer recovery (p=0.05). At six months, alcohol use (p=0.0282), smoking (p=0.0047), hemiparesis (p=0.0100), and high NIHSS scores remained significant predictors of unfavorable outcomes. At 12 months, the NIHSS score at presentation (p=0.0041) was the strongest predictor of long-term outcome. Other risk factors, including hypertension, diabetes, alcohol, and smoking, did not show statistically significant associations with unfavorable outcomes at one year (Table 6).

**Table 6:** Outcome analysis at thee, six, and 12 months by univariate analysis. NIHSS: National Institutes of Health Stroke Scale; HTN: hypertension; DM: diabetes mellitus

Variables	3 Months				6 Months					12 Months			
	Favorable (n=56)	Unfavorable (n=44)	Odds ratio	p-Value	Favorable (n=56)	Unfavorable (n=44)	Odds ratio	p-Value	Favorable (n=56)		Unfavorable (n=44)	Odds ratio	p-Value
Age (mean±SD)	55.22±10.8	55.09±10.1	1.0677	0.8892	55.44±11.2	54.60±13.6	0.996	0.8998	54.68±11.3		54.48±13.6	1.017	0.6402
Gender, n (%)	35 (62.5)	33 (75.0)	0.5556	0.1835	49 (65.3)	19 (76.0)	0.5951	0.3221	44 (66.7)		17 (75.0)	0.4706	0.2129
HTN, n (%)	47 (83.9)	35 (79.5)	1.3429	0.5712	61 (81.3)	21 (84.0)	0.8299	0.7638	51 (77.3)		18 (75.0)	0.5667	0.4055
DM, n (%)	27 (48.2)	25 (56.8)	0.7076	0.3926	35 (46.7)	17 (68.0)	0.4118	0.0645	35 (53.0)		14 (75.0)	0.5645	0.2725
Alcohol, n (%)	16 (28.6)	18 (40.9)	0.5778	0.1961	21 (28.0)	13 (52.0)	0.359	0.0282	22 (33.3)		11 (75.0)	0.4545	0.1171
Smoking, n (%)	13 (23.2)	20 (45.5)	0.2368	0.047	19 (25.3)	14 (56.0)	0.2666	0.0047	20 (30.3)		11 (75.0)	0.3953	0.0658
Altered consciousness, n (%)	6 (10.7)	7 (15.9)	0.5343	0.4432	7 (9.3)	6 (24.0)	0.326	0.059	8 (12.1)		5 (75.0)	0.4414	0.1907
Headache, n (%)	13 (23.21)	5 (11.36)	1.7286	0.3418	14 (18.7)	2 (8.0)	2.6393	0.2077	10 (15.2)		2 (75.0)	0.5667	0.4055
Dysarthria, n (%)	40 (71.4)	38 (86.4)	0.3947	0.0735	56 (74.7)	22 (88.0)	0.4019	0.1634	51 (77.3)		18 (75.0)	0.343	0.3646
Hemiparesis, n (%)	32 (57.1)	36 (81.8)	0.2963	0.0086	38 (50.6)	20 (75.0)	1.323	0.01	45 (68.2)		15 (75.0)	0.8571	0.7794
Monoparesis, n (%)	3 (5.4)	2 (4.5)	0.244	0.4569	3 (4.0)	2 (8.0)	0.4792	0.4268	2 (3.0)		2 (75.0)	0.2969	0.2159
Anterior circulation, n (%)	36 (64.2)	35 (79.5)	1.2374	0.439	49 (65.3)	18 (72.0)	0.7329	0.5393	43 (65.2)		15 (75.0)	0.7478	0.5951
Posterior circulation, n (%)	30 (53.6)	17 (38.6)	1.5326	0.1374	35 (46.7)	12 (48.0)	0.9479	0.9079	30 (45.5)		11 (75.0)	0.7576	0.5797
Infarct distribution, n (%)	48 (85.7)	36 (81.8)	1.3333	0.5978	19 (25.3)	7 (28.0)	0.8724	0.7924	10.82±0.7		11 (75.0)	0.257	0.5608
Follow-up duration (mean±SD)	11.46±1.1	11.29±1.7	0.609	0.0706	11.45±1.1	11.17±2.0	0.5876	0.3804	10.58±3.9		13.52±5.0	1.645	0.0041
NIHSS (mean±SD)	7.07±3.5	13.23±4.3	1.57	0.05	66 (80%)	18 (72%)	2.8519	0.0588	56 (84.8%)		15 (71.4%)	2.24	0.1668

## Discussion

Hypertension emerged as the most prevalent risk factor in this study, with 83.8% of males and 78.1% of females affected. Similar trends have been observed in other studies, where hypertension contributes significantly to vessel wall degeneration and atherosclerotic plaque formation [13,22]. The findings support previous studies that report no clear correlation between hypertension and the site of arterial occlusion. However, its role in altering cerebral perfusion and exacerbating atherosclerosis underscores its significance in stroke pathophysiology. Diabetes was frequently associated with posterior circulation strokes, notably in the vertebral (70.0%) and basilar (83.3%) arteries. Previous studies also indicate a higher prevalence of diabetes among patients with posterior circulation strokes [23]. Unlike anterior circulation strokes, which have multiple collateral pathways, posterior circulation infarcts often result in severe neurological deficits due to the limited collateral supply.

This study found no significant difference in hyperhomocysteinemia between males and females. However, hyperhomocysteinemia was more frequently associated with middle cerebral artery (MCA) involvement, aligning with previous studies where elevated homocysteine levels contributed to endothelial dysfunction and increased risk of MCA stenosis. The MCA was the most frequently affected artery (40%), followed by the internal carotid artery (11%) and the anterior cerebral artery (4%). This distribution is consistent with prior studies in Asian populations, where intracranial atherosclerosis predominantly affects the MCA. The lower prevalence of anterior cerebral artery involvement (4%) aligns with studies that highlight the protective effect of collateral circulation via the anterior communicating artery [24-26].

Posterior cerebral artery (PCA) involvement was observed in 15% of cases, often associated with vertebral or basilar artery lesions. The presence of concurrent basilar and vertebral artery stenosis suggests a high probability of artery-to-artery embolism, a well-documented mechanism in posterior circulation strokes [27]. Motor deficits were prominent in anterior circulation strokes, with hemiparesis occurring in 58% of cases. This aligns with infarcts affecting the motor cortex and subcortical structures supplied by the MCA and ICA. Hemianopia was observed in 45% of patients, reflecting infarcts in the temporoparietal cortex. In contrast, posterior circulation strokes were characterized by vertigo, unsteadiness, and visual field defects. Similar symptom distributions have been reported in stroke registries focusing on posterior circulation infarcts [28].

Seventeen patients experienced recurrent ischemic events, with the MCA (seven cases), ICA (four cases), and vertebrobasilar circulation (six cases) being the most common territories affected. Recurrence rates in this study (17%) were comparable to those reported in large stroke trials (12-23%). Notably, recurrence was significantly linked to the presence of three or more risk factors, emphasizing the importance of aggressive risk factor management. Uncontrolled hypertension and diabetes were significant contributors to recurrent stroke, with hypertension control reducing recurrence risk (p<0.05). Findings from previous studies suggest that post-stroke blood pressure management is critical in preventing further ischemic events. Similarly, poor glycemic control has been associated with early stroke recurrence, reinforcing the need for strict diabetes management in patients with intracranial atherosclerosis [29].

Patients on dual antiplatelet therapy had lower recurrence rates, while those on monotherapy showed a higher likelihood of stroke recurrence (p=0.004). The role of dual antiplatelet therapy in reducing nonfatal strokes and myocardial infarction has been well established [30]. Furthermore, patients on statin therapy demonstrated significantly lower recurrence rates (p=0.016), consistent with studies advocating intensive lipid-lowering therapy in patients with intracranial atherosclerosis. At three months, 56% of patients had a favorable outcome, whereas 44% had persistent deficits. Smoking was significantly linked to poor outcomes (p=0.047), reinforcing its role as a modifiable risk factor. At six months, alcohol use (p=0.0282) and hemiparesis (p=0.0100) were additional predictors of unfavorable outcomes. By 12 months, the NIHSS score at presentation (p=0.0041) remained the strongest predictor of long-term functional recovery. This study underscores the significance of hypertension, diabetes, and lifestyle-related factors in the recurrence and progression of intracranial atherosclerosis. The findings highlight the need for early risk factor modification and adherence to antiplatelet and statin therapy to improve long-term outcomes. Future research should focus on individualized treatment approaches and explore the role of emerging biomarkers in predicting stroke recurrence and severity. This study had a modest sample size, which may limit the generalizability of the findings. Although digital subtraction angiography (DSA) is the recognized gold standard for vascular imaging, its invasive nature and cost constraints make it less feasible for routine use. Instead, magnetic resonance angiography (MRA) was utilized, with reported diagnostic equivalency. While efforts were made to exclude patients with cardioembolic stroke, continuous cardiac monitoring, such as 24-hour Holter monitoring, was not performed in all patients, which may have influenced case classification. Additionally, patient-reported medication adherence was a limiting factor, as recall bias could have impacted accuracy. Patients with stenotic lesions who did not complete follow-up were excluded, which could introduce selection bias in outcome assessments. Non-contrast MRA was used, which has limitations due to blood flow artifacts that can mimic stenosis.

## Conclusions

Hypertension, diabetes mellitus, smoking, alcohol use, and hyperlipidemia were identified as significant risk factors for intracranial atherosclerosis-related stroke. Hypertension was the most observed modifiable factor. The study confirmed a higher prevalence of ischemic stroke due to intracranial atherosclerosis in males. The incidence of intracranial atherosclerotic disease was notably higher in the anterior circulation, with the MCA being the most frequently affected artery. High NIHSS scores, hemiparesis, smoking, and alcohol use were significant predictors of unfavorable outcomes. There was a considerable risk of early stroke recurrence in symptomatic intracranial artery stenosis. Patients on single antiplatelet therapy, those with uncontrolled hypertension, and those with poorly managed diabetes were at a higher risk of recurrent vascular events. Intensive medical management, including dual antiplatelet therapy, statins, and strict control of hypertension and diabetes, significantly reduced recurrence rates. Future studies in larger cohorts should validate these findings.
